# Non-operative anti-caries agents and dental caries increment among adults at high caries risk: a retrospective cohort study

**DOI:** 10.1186/s12903-015-0097-4

**Published:** 2015-09-24

**Authors:** Benjamin W. Chaffee, Jing Cheng, John DB Featherstone

**Affiliations:** Center to Address Disparities in Children’s Oral Health, UCSF School of Dentistry, 3333 California Street, Suite 495, San Francisco, CA 94143-1361 USA; UCSF School of Dentistry, 513 Parnassus Ave, Room S630, San Francisco, CA 94143-0430 USA

**Keywords:** Dental caries, Epidemiology, Caries management, Longitudinal studies, Health outcomes research

## Abstract

**Background:**

Consensus guidelines support non-operative preventives for dental caries management; yet, their use in practice is far from universal. The purpose of this study was to evaluate the effectiveness of non-operative anti-caries agents in caries prevention among high caries risk adults at a university clinic where risk-based caries management is emphasized.

**Methods:**

This retrospective observational study drew data from the electronic patient records of non-edentulous adult patients deemed to be at high risk for dental caries during baseline oral evaluations that were completed between July 1, 2007 and December 31, 2012 at a dental university in the United States. We calculated and compared adjusted mean estimates for the number of new decayed or restored teeth (DFT increment) from baseline to the next completed oral evaluation (*N* = 2,724 patients with follow-up) across three categories of delivery of non-operative anti-caries agents (e.g., high-concentration fluoride toothpaste, chlorhexidine rinse, xylitol products): never, at a single appointment, or at ≥2 appointments ≥4 weeks apart. Estimates were adjusted for patient and provider characteristics, baseline dental status, losses-to-follow-up, and follow-up time.

**Results:**

Approximately half the patients did not receive any form of non-operative anti-caries agent. Most that received anti-caries agents were given more than one type of product in combination. One-time delivery of anti-caries agents was associated with a similar DFT increment as receiving no such therapy (difference in increment: -0.04; 95 % CI: -0.28, 0.21). However, repeated, spaced delivery of anti-caries agents was associated with approximately one decayed or restored tooth prevented over 18 months for every three patients treated (difference in increment: -0.35; 95 % CI: -0.65, -0.08).

**Conclusions:**

These results lend evidence that repeatedly receiving anti-caries agents can reduce tooth decay among high-risk patients engaged in regular dental care.

**Electronic supplementary material:**

The online version of this article (doi:10.1186/s12903-015-0097-4) contains supplementary material, which is available to authorized users.

## Background

Despite long-standing consensus supporting minimal intervention and non-operative preventives for caries management in dental practice [1], a prevention-oriented strategy is far from reaching universal adoption: for example, many dentists favor restoration placement over non-operative therapy for enamel-confined lesions [2–4]. In contrast to traditional reliance on surgical means, a risk-based approach to the clinical management of dental caries stresses individualized treatment decisions based on patients’ behavioral and biological characteristics, with an emphasis on caries prevention and preservation of tooth structure [5–9].

Caries Management by Risk Assessment (CAMBRA) is one approach that has been proposed for patient-specific caries management [6]. First, in a risk assessment stage, the clinician is guided to categorize a patient’s caries risk based on an overall assessment of disease indicators, caries protective factors, and caries predisposing factors [6]. For adults categorized as high risk, CAMBRA clinical guidelines recommend providing antibacterial therapy (e.g., chlorhexidine or xylitol products) and remineralizing agents (e.g., high-concentration fluoride toothpaste) to manage caries as a disease process [10]. The CAMBRA approach has been firmly adopted in the university clinic in which this study was based.

Relatively few studies have evaluated the effectiveness of non-operative anti-caries management among adults at high caries risk. A recent randomized controlled trial reported that combined antibacterial and fluoride preventive therapy could lower caries risk and suggested a reduction in 2-year caries increment among initially high-risk patients [11]. The widespread implementation of electronic health records represents an opportunity to evaluate the effectiveness of personalized treatments in real practice [12, 13]. In two studies drawn from patient datasets, counseling adult patients at high caries risk to use fluoride toothpaste was associated with later classification into a lower risk category in one study [14], but in the other, fluoride therapy was not associated with significantly lower caries increment [15].

In the present retrospective observational study, we aimed to evaluate caries management outcomes based on electronic patient records at a university clinic where CAMBRA is emphasized: specifically, whether caries increment would be reduced among high-risk patients who received non-operative anti-caries agents. We hypothesized that among initially high-risk individuals, caries increment will be lowest among those patients who received non-operative anti-caries agents repeatedly over time.

## Methods

### Study design and population

This retrospective cohort study collected data from electronic patient records at the student dental clinic of the University of California San Francisco (UCSF). The UCSF Committee on Human Research (institutional review board) granted ethical approval for the use of retrospective patient data to evaluate patient outcomes according to existing clinical practices. The Committee did not require that explicit informed consent be collected for this investigation.

**Fig. 1:**
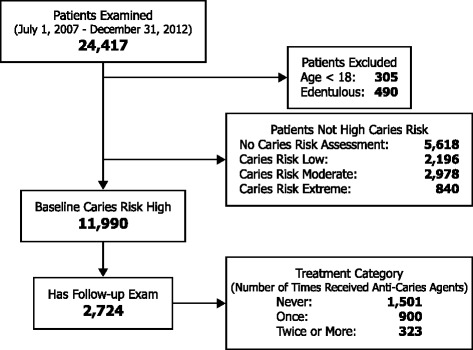
Flow diagram for participant inclusion criteria, treatment category, and follow-up. Among clinic patients that completed an oral examination during the study period, there were 2,724 eligible initially high-risk patients with a follow-up examination

Eligible for analysis were any patients who completed at least one full oral examination (new patient or recall) between July 1, 2007 and December 31, 2012 and were designated as high caries risk (Fig. 1). Excluded were any patients lacking teeth (third molars not counted) or less than 18 years of age. There were 11,990 high-risk patients fitting these criteria, of whom 2,724 completed at least one follow-up examination at least 180 days after baseline (Fig. 1).

We compared the number of newly decayed and restored teeth (DFT increment, a count outcome) and caries incidence (DFT increment >0, a dichotomous outcome for having any newly affected teeth *versus* none) from baseline to follow-up across three levels of delivery of non-operative anti-caries agents (e.g., fluoride, chlorhexidine, and/or xylitol products): never, at a single clinic visit, or at ≥2 visits ≥4 weeks apart.

### Study variables

Patients designated as high caries risk were included in this study. Student providers, under the guidance of faculty dentists, had assigned baseline caries risk categories following CAMBRA guidelines, which were developed by an expert working group of clinicians and research scientists [6, 16]. In the caries risk assessment component of CAMBRA, a clinician classifies a patient’s caries risk (i.e., low, moderate, high, or extreme) after consideration of the balance between existing biological predisposing factors (e.g., frequent between meal snacking, heavy plaque on teeth, reduced saliva flow), caries protective factors (e.g., fluoride exposure, use of chlorhexidine rinse), and disease indicators (e.g., cavitated lesions, recently placed restorations), collected on a standard caries risk assessment form [6]. No rigid classification algorithm is used. Rather, the clinician assesses the overall balance of predisposing and protective factors. The CAMBRA risk assessment approach has been shown to stratify patients into risk categories of increasing future caries activity, both among adults in a university clinic [17] and among kindergarten children in Hong Kong [18]. Extreme-risk patients were excluded from the present analysis due to the relatively small number of patients assigned this risk designation.

DFT increment was the number of teeth (non-third molars) between baseline and follow-up recorded as having new carious decay (excluding white spot, arrested, and enamel-confined lesions, but including decay around existing restorations and root caries) or new restorations (i.e., amalgam, composite, glass ionomer, onlay/inlay, or crown, but excluding restorations placed on teeth affected by abfraction, attrition, or erosion without caries involvement), as retrieved from electronic patient records using practice management software (axiUm, Exan Group, Vancouver, Canada). Teeth with planned restorative treatment or decay at baseline were not counted in the DFT increment. To avoid including teeth needing treatment only for periodontal, orthodontic, or esthetic reasons, we excluded teeth designated for extraction and veneers or crowns placed on anterior teeth, unless caries was recorded. We made the reasonable assumption that restorative treatment completed within 180 days of baseline was in response to baseline decay rather than new conditions for inclusion in DFT increment. The threshold 180 days was chosen based on observed patterns in the timing of treatments in the student clinic and has been used elsewhere [15]. Analogously, we did include restorations placed up to 180 days after follow-up in calculating DFT increment.

We defined three categories of non-operative anti-caries therapy. Anti-caries therapy was defined as receiving any anti-caries agent between baseline and follow-up, including chlorhexidine rinse (0.12 % chlorhexidine gluconate), topical fluoride (e.g., fluoride toothpaste at 5000 ppm F or fluoride varnish), and xylitol products (e.g., mint-flavored tablets). Clinic procedures recommend the use of a combination of these products for high-risk individuals. Categories for data analysis and comparison were: 1) none (anti-caries agents never received); 2) one time (received at a single patient visit, even if multiple agents were dispensed); and 3) two or more times (≥2 visits, ≥4 weeks apart). Patients that received anti-caries agents at more than one visit but within a single 4-week period and who did not received anti-caries agents again were categorized as one-time delivery. We selected these categories to approximate never use, one-off dispensing, and continuing use of anti-caries agents.

Confounding variables included baseline patient characteristics: age (categorized as 18–34, 35–44, 45–54, 55–64, and ≥65 years), sex, payer type (private dental insurance, public dental benefits, or no insurance/cash), self-identified race/ethnicity (African American, Asian, Caucasian, Hispanic/Latino, or other/declined to state), number of teeth; and student provider characteristics: program (4-year doctoral program or 2-year program for internationally trained dentists) and year in training (final year or next-last year). We also adjusted for number of decayed teeth at baseline, because baseline disease status may have influenced the decision to pursue non-operative therapy. As a secondary analysis, we also included patient characteristics recorded in the baseline risk assessment form (all binary variables): radiographic or visible dentin cavitation, heavy dental plaque, frequent snacking (>3 times daily between meals), twice-daily fluoride toothpaste, adequate saliva flow, and living, working, or attending school in a fluoridated community.

We performed an exploratory subgroup analysis, in which we calculated the difference in DFT increment according to category of non-operative anti-caries therapy in different patient groups. We repeated the analysis to calculate caries outcomes but restricted the population to subgroups defined according to patient sex, payer type (private dental insurance, public dental benefits, no insurance/cash), and age (18–44 years, ≥45 years).

## Statistical power

The sample size used in the main analysis was a result of the number of eligible high-risk patients attending the clinic from 2007–2012. Given 1,501 patients with follow-up who received no anti-caries therapy and 323 who received therapy twice or more, the study would have 80 % power to detect a 0.26 reduction in DFT increment and 90 % power to detect a 0.30 reduction, assuming 1.75 DFT increment in the no therapy group (standard deviation = 1.5, alpha threshold for statistical significance = 0.05, two-tailed test). The study would have 80 % power to detect a 0.87 ratio in DFT increment >0 between the two groups and 90 % power to detect a ratio of 0.85, assuming 65 % of patients with DFT increment >0 in the no therapy group (alpha = 0.05, two-tailed test).

## Statistical approach

We calculated doubly-robust adjusted estimates for caries outcomes according to categories of anti-caries therapy using g-computation and inverse probability treatment weighting in a combined approach [19]. This technique has been described approachably in recent publications [20, 21]. We fitted regression models for caries outcomes (negative binomial model for DFT increment and logistic model for DFT increment >0), where anti-caries therapy category was the exposure variable and baseline covariates were age, sex, race/ethnicity, payer type, baseline number of teeth, baseline number of decayed teeth, calendar year, provider program, provider years in training, and follow-up time. Models were used to predict adjusted marginal caries outcomes under each category of anti-caries therapy received, setting follow-up time to 18-months (548 days), the mean value in the follow-up sample. Regression models were weighted using inverse probability treatment weights to enhance robustness to model misspecification and using inverse probability censoring weights to account for losses to follow-up from the baseline sample [19, 22]. We multiply imputed missing baseline data (0.2 % of covariate data among eligible participants) and averaged point estimates over 25 imputations. Results were unchanged in a sensitivity analysis restricted to cases with complete baseline covariate data.

Estimates represent the expected DFT increment associated with each level of anti-caries therapy under the same distribution of participant characteristics that was observed in the baseline population and with equal follow-up time (18 months). As measures of association, we computed the difference in DFT increment and ratio in the percentage of patients with DFT increment >0, given single or repeated delivery of anti-caries therapy, as two separate pair-wise comparisons, each with respect to no therapy received. We used the percentile bootstrap method (3000 bootstrap re-samples) to obtain 95 % confidence intervals (CI) and considered results to be statistically significant at the 0.05 level if the 95 % CI for measures of association excluded the null value. Analyses were performed using statistical software (Stata 13.1, StataCorp LP, College Station, United States and R 3.1.2, R Foundation for Statistical Computing, Vienna, Austria). Study reporting followed the STROBE statement [23] (Additional file 1) .

## Results

Of the 11,990 patients categorized as high caries risk at baseline in this clinic, nearly two-thirds lacked either public or private dental benefits coverage, and most racially/ethnically identified as non-Caucasian. Mean patient age was 46.2 years (SD: 17.0; range: 18–99). Patients who completed at least one follow-up visit were more likely to be male, in a more mature age category, identify as Caucasian, and have private or public dental benefits than patients who did not complete any follow-up visit (all *P* < 0.001; chi-square test); however, the baseline and follow-up samples were practically similar in the overall distributions of measured demographic characteristics (Table 1). Mean follow-up time was 542 days.

**Table 1:** Study population characteristics, by follow-up status

Characteristic	Baseline sample	Sample lost to follow-up	Sample with follow-up
	*N* = 11,990	*N* = 9,266	*N* = 2,724
Patient sex, %			
Male	48.1	47.2	51.2
Female	51.9	52.8	48.8
Patient age, %			
18–34 years	32.0	34.8	22.4
35–44 years	16.4	17.1	14.0
45–54 years	17.9	17.7	18.9
55–64 years	17.2	16.1	20.9
≥65 years	16.4	14.3	23.8
Patient payer type, %			
Private insurance	14.8	13.8	18.1
Public program	21.0	20.1	24.3
Cash	64.2	66.1	57.6
Patient race/ethnicity, %			
African American	10.5	10.7	9.5
Asian	13.6	13.5	13.9
Caucasian	43.4	41.8	49.1
Hispanic/Latino	17.8	18.3	15.9
Other or declined to state	14.7	15.7	11.5
Provider type, %			
Doctoral 4-year program	77.7	77.8	77.5
International 2-year program	22.3	22.2	22.5
Provider year of training, %			
Final year	47.9	47.5	49.2
Next-to-last year	52.1	52.5	50.8

Among the 2,724 patients with a follow-up examination, 55.1 % did not receive any non-operative anti-caries agent (Fig. 1). Of those who did receive some form of non-operative anti-caries agent, 68.8 % began therapy within 30 days of the baseline visit and all patients began therapy within 180 days of the baseline visit. Of the 323 patients who received anti-caries agents at two or more visits, 82.7 % were given more than one type of agent. In this group, 83.0 % received high-concentration (5000 ppm F) fluoride gel or toothpaste for home use, 67.2 % received chlorhexidine rinse (0.12 % chlorhexidine gluconate), and 42.7 % received xylitol-containing products (e.g., lozenges, mints, or chewing gum). Those patients who received anti-caries agents twice or more were more likely to be female, older, and to receive dental benefits through a public program (Table 2).

**Table 2:** Study population characteristics and caries-related factors, by anti-caries treatment provided between baseline and follow-up

Characteristic	Received anti-caries agents never	Received anti-caries agents once	Received anti-caries agents twice or more
	*N* = 1,501	*N* = 900	*N* = 323
Patient sex, %			
Male	52.6	50.5	46.3
Female	47.4	49.5	53.7
Patient age, %			
18–44 years	37.1	38.2	27.2
≥45 years	62.9	61.8	72.8
Patient payer type, %			
Private insurance	19.5	17.4	13.6
Public program	20.1	23.7	45.5
Cash	60.4	58.9	40.9
Patient race/ethnicity, %			
African American	10.4	8.1	9.6
Asian	13.8	13.8	14.9
Caucasian	45.6	53.3	53.6
Hispanic/Latino	17.0	14.8	14.2
Other or declined to state	13.3	10.0	7.7
Provider type, %			
Domestic 4-year program	73.0	82.4	84.5
International 2-year program	27.0	17.6	15.5
Provider year of training, %			
Final year	50.6	48.2	45.2
Penultimate year	49.4	51.8	54.8
Radiographic or visible dentin cavitation (baseline), %	45.3	44.7	41.8
Heavy plaque, %	59.1	56.1	58.4
Frequent snacking, %	37.0	36.4	38.7
Fluoride toothpaste, twice daily, %	71.1	73.7	76.2
Adequate saliva flow, %	80.2	81.7	77.6
Lives/works in fluoridated community, %	81.2	79.3	83.5

Percentages listed in Table 2 exclude missing data. Extent of missing values was lower for variables used in analysis (age: 0 %, race/ethnicity: 0 %, provider type: 0 %, provider year of training: 0 %, payer type: 0.3 %, and sex: 0.4 %) than for descriptive caries-risk variables (e.g., 36.5 % for radiographic or visible dentin cavitation)

Among initially high-risk patients, receipt of anti-caries agents at two or more appointments was associated with a statistically significant 19 % reduction in adjusted DFT increment over 18 months: 1.47 affected teeth compared to 1.82 affected teeth in the no therapy group (Table 3). However, one-time receipt of anti-caries agents was not associated with a meaningful difference in DFT increment compared to the group that received no anti-caries agents (Table 3). Unlike DFT increment, the percentage of patients with any new decay (DFT increment >0) did not differ substantially across the three groups, regardless of whether anti-caries agents were received once or repeatedly (Table 3). These results were not altered substantially in a secondary analysis that adjusted for additional baseline caries risk factors, preventive factors, and disease indicators: in this analysis, the difference in DFT increment between receipt of anti-caries agents at two or more visits *versus* none was -0.32 (95 % CI: -0.64, -0.02).

**Table 3:** Caries increment from baseline to follow-up examination among baseline high-risk patients, by receipt of anti-caries agents

Anti-caries agent (s) received	DFT Increment, observed	DFT Increment, adjusted^a^	DFT Difference^a^(95 % CI)	Caries Incidence (DFT > 0), observed, %	Caries Incidence (DFT > 0), adjusted^a^, %	Caries Incidence Risk Ratio^a^ (95 % CI)
Not received	1.76	1.82	reference	62.5	64.6	reference
Once	1.74	1.78	−0.04 (−0.29, 0.20)	62.8	64.2	0.99 (0.90, 1.07)
Twice or more	1.62	1.47	−0.35 (−0.65, −0.08)	61.3	62.5	0.97 (0.82, 1.06)

Abbreviations: *CI* confidence interval, *DFT* decayed, restored tooth index

Results based on 2,724 high caries risk patients with baseline caries risk assessment from 2007–2012

^a^Adjusted models account for patient age, sex, payer type, race/ethnicity, provider type and year in training, calendar year, baseline decayed teeth, baseline number of teeth, losses-to-follow-up, and follow-up time

**Fig. 2:**
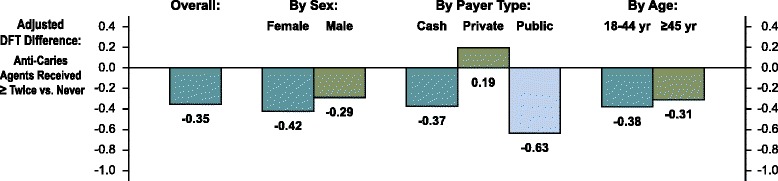
Subgroup results. The figure demonstrates the adjusted 18-month difference in the number of decayed or restored teeth between patients who received non-operative anti-caries agents repeatedly *versus* none received, according to subgroups defined by patient characteristics

In an exploratory subgroup analysis, we assessed whether the difference in DFT increment between receiving anti-caries agents twice or more and receiving no anti-caries agents varied by patient characteristics (Fig. 2). Notably, the reduction in DFT increment with repeated anti-caries therapy was greatest among patients with public dental benefits (difference in increment: -0.63), a group that almost entirely comprised patients enrolled in the state Medicaid dental program and who were offered anti-caries products at no charge.

## Discussion

This study was one of the few to evaluate caries outcomes among high-risk adults following delivery of non-operative preventives, and to our knowledge, the first study to examine outcomes according to the frequency with which preventives were received. Delivery of non-operative anti-caries agents at multiple visits was associated with a 19 % reduction in DFT increment but no statistically significant difference in the incidence of new decay (DFT increment >0). This indicates that in high-risk populations, anti-caries agents might be most useful for reducing disease severity among caries affected individuals, as opposed to preventing caries entirely. In a recently reported randomized controlled trial of high caries risk adults, a combined antibacterial and fluoride therapy intervention resulted in no difference in the probability of having no incremental lesions in a zero-inflated Poisson model but yielded a statistically significant 24 % decrease in DMFS increment for the count portion of the model [11]. Similarly, a community randomized trial featuring fluoride varnish applications for Aboriginal children in Australia reported a prevented fraction for incremental lesions of approximately 30 % but no statistically significant change in the prevalence of children with ≥1 affected teeth [24].

In a similar retrospective study of administrative data from two large US dental health plans, in one plan, a formal recommendation for at-home fluoride treatment for high-risk patients was associated with a non-statistically significant 11 % reduction in caries increment [15]. However, in the second dental plan, high-risk patients who received in-office topical fluoride demonstrated higher caries increment than high-risk patients given no therapy [15]. The authors speculated that dentists in this plan “further stratified” patients within the high risk category, choosing to deliver preventive therapy more often to a subset of high-risk patients that they deemed even more likely to experience future decay [15]. In the present study, we attempted to account for such confounding by indication by adjusting for the number of decayed teeth at baseline, and in the adjusted analysis, we did observe a statistically significant difference in DFT increment according to therapy received.

While receipt of anti-caries agents at multiple visits was associated with lower caries incidence, equivalent to slightly more than one decayed/restored tooth prevented over 18 months for every three patients treated, more than half of these high-risk patients did not receive any form of anti-caries agent. Whether less than universal use of non-operative therapies reflects reticence on the part of providers, patients, or both, our results suggest that greater prevention could be achieved if non-operative therapies were more widely utilized.

Notably, in the exploratory subgroup analysis, having dental benefits through a public program was associated with the greatest reduction in DFT increment with repeated delivery of anti-caries agents. A special arrangement between the dental school and the administrator of the state Medicaid dental program made it possible for the university clinic to collect reimbursement for risk-based preventive treatments and to provide them at no charge to the patients who agreed to accept them. Therefore, it is plausible that this reimbursement mechanism eased the way for more intensive preventive therapy, both in terms of the frequency of delivery and the number of different types of products provided, potentially leading to a larger impact among these patients.

We had no measure of patient adherence to recommended regimens for home-use anti-caries agents. We speculate that poor patient adherence accounts for the lack of anti-caries effectiveness associated with one-time therapy. In contrast, we hypothesize that patients who were dispensed agents on multiple occasions reflect adherence patterns consistent with continuing home-use and return for agent replenishment. The vast majority of patients who received anti-caries agents on multiple occasions were given more than one type of agent (fluoride, xylitol, or chlorhexidine), which follows the documented protocol emphasized in this clinic. Thus, it was not possible to determine if any one agent was most effective. The CAMBRA approach, which aims both to decrease pathological factors (antibacterial therapy) and simultaneously to enhance preventive or reparative therapy (e.g., via high concentration fluoride product), likely operates through multiple mechanistic pathways [25].

Harnessing routinely collected data from electronic health records for clinical research presents challenges but also promises to expand clinical research capacity [26]. Data analyzed in this study were not collected specifically for use in research. For example, student providers did not undergo a specific calibration exercise in caries detection, and there was no rigid methodology applied to treatment planning or caries risk assessment, although all providers were part of the same educational program, which teaches and emphasizes CAMBRA, and at the time of this study, allowed for radiographic, visual, and tactile methods to be used in caries detection. Also, it is possible that our calculation of the DFT increment included some restorations that were placed for reasons other than dental caries, leading to an overestimation of caries occurrence in all comparison groups. Such limitations were partly balanced by access to a large analytic sample that reflects realistic treatment decisions made outside the context of a formal intervention study.

Further research is required to determine whether the results observed in this study can be generalized beyond this educational clinic, which predominantly serves lower-income patients at high caries risk and in which dental students are primarily responsible for diagnostic and preventive care. Additionally, most patients observed at baseline did not return to the clinic for a follow-up examination, which could also decrease the generalizeability of our findings. However, we implemented inverse probability censoring weighting to account for differences in measured characteristics between the baseline and follow-up samples. We did not evaluate therapy outcomes among low, moderate, or extreme risk patients due to the smaller number of patients in these categories, particularly in the extreme risk group: a category marked by severe hypo-salivation and for which guidelines suggest intensive preventive care in multiple forms [10], which may surpass the level of prevention provided to high-risk patients in this study. Furthermore, consistent with CAMBRA guidelines, relatively few low- and moderate-risk patients received anti-caries agents.

We obtained adjusted outcome values through implementation of a doubly-robust version of the g-computation estimator, a technique rarely applied in oral health research, despite increasingly common use in epidemiology, generally [27, 28]. An attractive aspect of this approach is the ease of interpretation: estimates take the form of expected caries outcomes associated with each category of interest under equal covariate distributions. However, as with all observational studies, analyses must account for confounding variables, and it is possible that unmeasured factors could have affected the results.

## Conclusions

This study represents one of the largest longitudinal evaluations of clinical caries outcomes following risk-based non-operative therapy. These findings suggest that aggressive management with remineralization and/or antibacterial agents can successfully reduce the severity of dental caries in high-risk patients and supports the use of such agents in caries management among individuals seeking dental care.

## Additional file

Additional file 1:
**STROBE Statement—Checklist of items that should be included in reports of cohort studies.** (DOC 82 kb)

## Abbreviations

CAMBRA: Caries management by risk assessment
CI: Confidence interval
DFT: Number of decayed or restored teeth
ppm F: Parts per million fluoride ion
SD: Standard deviation
UCSF: University of California San Francisco
US: United States
