# Supplementary material for: Non-operative anti-caries agents and dental caries increment among adults at high caries risk: a retrospective cohort study
Source: BMC Oral Health. 2015 Sep 24;15:111. doi: 10.1186/s12903-015-0097-4 (PMC4581405; doi:10.1186/s12903-015-0097-4)
Supplement: Additional file 1: — STROBE Statement—Checklist of items that should be included in reports of cohort studies. (DOC 82 kb) [file 12903_2015_97_MOESM1_ESM.doc]

STROBE Statement—Checklist of items that should be included in reports of ***cohort studies***

|  | Item No | Recommendation |
| --- | --- | --- |
| **Title and abstract** | 1 | (*a*) Indicate the study’s design with a commonly used term in the title or the abstract  in title (page 1) and abstract (page 2) |
|  |  | (*b*) Provide in the abstract an informative and balanced summary of what was done and what was found  abstract - page 2 |
| Introduction | | |
| Background/rationale | 2 | Explain the scientific background and rationale for the investigation being reported  background - page 3 |
| Objectives | 3 | State specific objectives, including any prespecified hypotheses  background - page 4 (lines 6-12) |
| Methods | | |
| Study design | 4 | Present key elements of study design early in the paper  methods - pages 4 (design), page 5 (population + key variables) |
| Setting | 5 | Describe the setting, locations, and relevant dates, including periods of recruitment, exposure, follow-up, and data collection  methods - page 4 (location + dates), pages 5-6 (periods of exposure + follow-up) |
| Participants | 6 | (*a*) Give the eligibility criteria, and the sources and methods of selection of participants. Describe methods of follow-up  methods - page 4 (eligibility); selection + follow-up (N/A = retrospective) |
|  |  | (*b*) For matched studies, give matching criteria and number of exposed and unexposed  N/A = not matched |
| Variables | 7 | Clearly define all outcomes, exposures, predictors, potential confounders, and effect modifiers. Give diagnostic criteria, if applicable  methods - pages 5-7 (study variables) |
| Data sources/ measurement | 8* | For each variable of interest, give sources of data and details of methods of assessment (measurement). Describe comparability of assessment methods if there is more than one group  methods - pages 5-7 (study variables) |
| Bias | 9 | Describe any efforts to address potential sources of bias  methods - page 8 (adjustment for confounding, missing data, + losses to follow-up) |
| Study size | 10 | Explain how the study size was arrived at  methods - pages 7 +8 (statistical power) |
| Quantitative variables | 11 | Explain how quantitative variables were handled in the analyses. If applicable, describe which groupings were chosen and why  methods - pages 5-7 (study variables), page 8 (statistical approach) |
| Statistical methods | 12 | (*a*) Describe all statistical methods, including those used to control for confounding  methods - page 8 (statistical approach) |
|  |  | (*b*) Describe any methods used to examine subgroups and interactions  methods - page 7 (lines 15-19: subgroup analysis) |
|  |  | (*c*) Explain how missing data were addressed  methods - page 8 (statistical approach: multiple imputation) |
|  |  | (*d*) If applicable, explain how loss to follow-up was addressed  methods - page 8 (statistical approach: inverse probability of censoring weights) |
|  |  | (*e*) Describe any sensitivity analyses  methods - page 8 (statistical approach: complete case sens. analysis) |
| Results | | |
| Participants | 13* | (a) Report numbers of individuals at each stage of study—eg numbers potentially eligible, examined for eligibility, confirmed eligible, included in the study, completing follow-up, and analysed  Figure 1 - flow diagram with N at each stage |
|  |  | (b) Give reasons for non-participation at each stage  N/A in retrospective study: individuals are not “participants” at time data are collected |
|  |  | (c) Consider use of a flow diagram  Figure 1 |
| Descriptive data | 14* | (a) Give characteristics of study participants (eg demographic, clinical, social) and information on exposures and potential confounders  Table 1 + Table 2 |
|  |  | (b) Indicate number of participants with missing data for each variable of interest  Table 2 - legend |
|  |  | (c) Summarise follow-up time (eg, average and total amount)  Page 8: lines 15-16 |
| Outcome data | 15* | Report numbers of outcome events or summary measures over time  Table 3 |
| Main results | 16 | (*a*) Give unadjusted estimates and, if applicable, confounder-adjusted estimates and their precision (eg, 95% confidence interval). Make clear which confounders were adjusted for and why they were included  Table 3 |
|  |  | (*b*) Report category boundaries when continuous variables were categorized  Page 7: lines 3-14 |
|  |  | (*c*) If relevant, consider translating estimates of relative risk into absolute risk for a meaningful time period  Table 3 |
| Other analyses | 17 | Report other analyses done—eg analyses of subgroups and interactions, and sensitivity analyses  Figure 2 |
| Discussion | | |
| Key results | 18 | Summarise key results with reference to study objectives  Discussion: page 11, lines 1-15 |
| Limitations | 19 | Discuss limitations of the study, taking into account sources of potential bias or imprecision. Discuss both direction and magnitude of any potential bias  Discussion: pages 11-14 |
| Interpretation | 20 | Give a cautious overall interpretation of results considering objectives, limitations, multiplicity of analyses, results from similar studies, and other relevant evidence  Discussion: pages 11-14 |
| Generalisability | 21 | Discuss the generalisability (external validity) of the study results  Discussion: page 13 |
| Other information | | |
| Funding | 22 | Give the source of funding and the role of the funders for the present study and, if applicable, for the original study on which the present article is based  Aknowledgements: page 15 |

*Give information separately for exposed and unexposed groups.

**Note:** An Explanation and Elaboration article discusses each checklist item and gives methodological background and published examples of transparent reporting. The STROBE checklist is best used in conjunction with this article (freely available on the Web sites of PLoS Medicine at http://www.plosmedicine.org/, Annals of Internal Medicine at http://www.annals.org/, and Epidemiology at http://www.epidem.com/). Information on the STROBE Initiative is available at http://www.strobe-statement.org.
